# Hemosiderin-Laden Macrophages in Bronchoalveolar Lavage: Predictive Role for Acute Exacerbation of Idiopathic Interstitial Pneumonias

**DOI:** 10.1155/2021/4595019

**Published:** 2021-12-20

**Authors:** Toru Arai, Tomoko Kagawa, Yumiko Sasaki, Reiko Sugawara, Chikatoshi Sugimoto, Kazunobu Tachibana, Yoshiki Fujita, Seiji Hayashi, Yoshikazu Inoue

**Affiliations:** ^1^Clinical Research Center, National Hospital Organization Kinki-Chuo Chest Medical Center, Sakai City, Osaka, Japan; ^2^Department of Internal Medicine, National Hospital Organization Kinki-Chuo Chest Medical Center, Sakai City, Osaka, Japan; ^3^Department of Respiratory Medicine, Gifu Prefectural Tajimi Hospital, Tajimi City, Gifu, Japan; ^4^Department of Laboratory Medicine, National Hospital Organization Hyogo-Chuo National Hospital, Sanda City, Hyogo, Japan; ^5^Department of Internal Medicine, Aihara Dai-ni Hospital, Osaka City, Osaka, Japan

## Abstract

**Background:**

Hemosiderin-laden macrophages (HLMs) have been identified in the bronchoalveolar lavage fluid (BALF) of patients with idiopathic pulmonary fibrosis (IPF). This retrospective study examined the ability of HLMs in BALF to predict the acute exacerbation (AE) of chronic idiopathic interstitial pneumonias (IIPs).

**Methods:**

Two hundred and twenty-one patients with IIP diagnosed by bronchoscopy were enrolled in the study (IPF, *n* = 87; IIPs other than IPF, *n* = 134). Giemsa stain was used to detect HLMs in BALF specimens. Prussian blue stain was used to quantify HLMs in BALF, and a hemosiderin score (HS) was given to the specimens containing HLMs.

**Results:**

Twenty-four patients had a positive HS (range: 7‒132). The receiver-operating characteristic curve analysis identified the cutoff HS value for predicting the AE of IIPs to be 61.5. Seven cases had a higher HS (≥61.5) and 214 had a lower HS. AE occurred significantly earlier in the higher HS group (4/7 cases) than in the lower HS group (41/214 cases) during a median observation period of 1239 days (log-rank test, *p* = 0.026). Multivariate Cox proportional hazard regression analysis showed that a higher HS was a significant predictor of AE in addition to IPF, percent predicted forced vital capacity, and modified Medical Research Council score. The C-statistics for the prediction of AE did not significantly improve by all the above parameters with HS as compared without HS.

**Conclusions:**

A higher HS was a significant predictor of AE in IIPs but did not significantly improve the predictive ability of other parameters.

## 1. Introduction

Idiopathic pulmonary fibrosis (IPF) is a fibrotic lung disease with a usual interstitial pneumonia (UIP) pattern, a poor prognosis, and an unknown etiology [1, 2]. IPF is characterized by the progressive worsening of dyspnea and lung function, however, some patients with IPF experience rapid, and often, fatal deterioration [3–7]. These episodes, known as the acute exacerbations (AEs) of IPF (AE-IPF), are often of unclear etiology. The AEs of fibrotic lung disease were originally reported in IPF but have since been documented in various types of idiopathic interstitial pneumonias (IIPs) [8, 9]. Our group has recently described the frequency and prognosis of AE in IIPs (AE-IIPs) [9, 10].

Iron deposition in the lung can be caused by exogenous factors, including smoking [11] and dust exposure, and endogenous factors, such as occult hemorrhage [12]. The transformation of excess iron into hemosiderin by macrophages is a mechanism that attenuates iron-induced oxidative stress and its inflammatory and fibrogenic effects. Therefore, an increase in the hemosiderin-laden macrophage (HLM) level in bronchoalveolar lavage fluid (BALF) is assumed to reflect the excessive iron deposition in the lungs [12].

Diffuse alveolar hemorrhage can be diagnosed by the quantification of HLMs in BALF [13]. Golde et al. devised a hemosiderin score (HS) that is reportedly useful for the diagnosis of alveolar hemorrhage, whether idiopathic or infectious in origin [13]. In patients with acute respiratory distress syndrome (ARDS), which is characterized pathologically by diffuse alveolar damage, a higher HS at the time of diagnosis heralds a poor prognosis [14].

Several studies have identified a greater deposition of hemosiderin and higher HLM levels in BALF in the patients with IPF [12, 15] than in controls. Puxeddu et al. found that the HLM level in BALF was significantly higher in the patients with IPF than in controls and that a smoking history had no significant effect on the numbers of HLMs [12]. Furthermore, a significant correlation was found between the HLM levels in BALF and pulmonary hypertension (PH) detected by echocardiography [16] or right heart catheterization [17] in patients with IPF.

A higher modified Medical Research Council (mMRC) score for shortness of breath and a lower forced vital capacity (FVC) are the known predictors of AE-IPF [18] and AE-IIP [9]. The neutrophil counts in BALF are known to increase during AE-IPF [19], and the neutrophil level in BALF at the time of the diagnosis of IPF is a significant predictor of AE-IPF and AE-IIP [9]. Furthermore, the activation of macrophages is thought to be associated with AE-IPF [20]. In chronic obstructive pulmonary diseases, the percentage of positive HLMs in sputum was associated with the number of AEs in the previous 2 years [21]. Therefore, we hypothesized that HLMs in BALF would predict AE-IIPs.

The aim of this single-center observational study was to determine whether HLMs in BALF in the diagnosis of IIP can predict the subsequent AE and the ability of multivariate models with/without HLMs to predict AE-IIP.

## 2. Patients and Methods

A search of the National Hospital Organization Kinki-Chuo Chest Medical Center database of bronchoalveolar lavage (BAL) for 2005–2009 identified 231 consecutive cases of IIP with or without transbronchial lung biopsy. Bronchoscopy is performed at our institution when IIP is suspected, provided the patient can tolerate the pulmonary function tests. BAL was performed before treatment in all cases [9].

**Figure 1:**
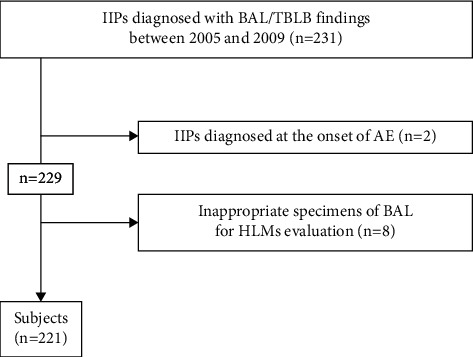
Flow chart of subject selection. Abbreviations: IIPs, idiopathic interstitial pneumonias; BAL, bronchoalveolar lavage; TBLB, transbronchial lung biopsy; AE, acute exacerbation; HLMs, hemosiderin-laden macrophages.

Two patients who had been found to have AE at the time of the initial diagnosis of IIP were excluded [9]. Eight further patients were excluded because their BALF specimens could not be evaluated by Prussian blue stain. Finally, 221 patients with IIPs (IPF, *n* = 87; non-IPF, *n* = 134) were enrolled (Figure 1). Most of these cases have been reported previously [9]. IPF was diagnosed according to the American Thoracic Society (ATS)/European Respiratory Society (ERS)/Japanese Respiratory Society (JRS)/Latin American Thoracic Association guideline [2]. IIPs were diagnosed according to the ATS/ERS statement [22]. IIPs other than IPF, including those diagnosed by surgical lung biopsy (SLB) [22] and those that were unclassifiable without SLB, were classified as non-IPF (Table 1). IIPs found histologically to be pleuroparenchymal fibroelastosis with UIP were diagnosed as IPF (*n* = 2). Four cases of IPF diagnosed from specimens obtained during surgery for lung cancer were included in the cases diagnosed by SLB.

High-resolution computed tomography scans were independently reviewed by the same chest radiologist who was blinded to the clinical findings. Based on the pattern observed, the cases were classified according to the IPF guidelines [2] as “UIP” (*n* = 59), “possible UIP” (*n* = 97), or “inconsistent with UIP” (*n* = 65).

The study protocol was approved by the Kinki-Chuo Chest Medical Center review board (approval number 463; date of approval, May 5, 2014). The requirement for informed consent was waived in view of the retrospective observational nature of the research and the anonymity of the data.

### 2.1. HLMs in BALF at the Time of Diagnosis of IIPs

BAL was performed using three 50-ml-aliquots of saline as described elsewhere [23]. Giemsa stain was used for the analysis of cells and detection of hemosiderin in BALF specimens. Hemosiderin-positive specimens were then stained with Prussian blue to quantify HLMs in BALF using a modified version of the HS described by Golde [13]. The hemosiderin content of 500 alveolar macrophages was graded 0–4, and the sum of grades was divided by 5, corresponding to the sum of 100 cells. Each slide was scored three times, with the average providing the modified HS. Cases that were hemosiderin-negative by Giemsa staining were given an HS of 0.

### 2.2. Diagnosis of AE-IIPs

AE-IIPs were diagnosed based on the following modified Japanese Respiratory Society criteria [24]: (1) within one month after the chronic clinical course of IIPs, the following three conditions are satisfied: (i) progressively worsening dyspnea, (ii) new ground-glass opacities evident on high-resolution computed tomography scans superimposed on a background reticular or honeycomb pattern, and (iii) a reduction in resting the partial pressure of oxygen in arterial blood (PaO_2_) by more than 10 Torr compared with previous measurements and (2) the obvious causes of acutely impaired respiratory function, such as infection, pneumothorax, cancer, pulmonary embolism, or congestive cardiac failure, are excluded.

### 2.3. Clinical Findings at Diagnosis

Demographic and clinical data, including age, sex, body mass index (BMI), smoking status, mMRC scores [25], pulmonary function tests, and serum markers at the time of diagnosis of the IIPs, were obtained from the medical records. The pulmonary function tests were performed using a Chestac 8080 device (CHEST M.I., INC., Tokyo, Japan).

### 2.4. Measurements of Serum Markers

Serum Krebs von den Lungen (KL)-6 and surfactant protein (SP)-D levels were measured using commercially available enzyme-linked immunosorbent assay kits (KL-6: Eizai, Tokyo, Japan; SP-D: Kyowa Medex, Tokyo, Japan). The KL-6 and SP-D cutoff levels were 500 U/mL and 110 ng/mL, respectively [26].

### 2.5. Histological Findings in SLB Specimens

SLB specimens with a higher HS, described in the following section, were evaluated for the presence of HLMs and vascular lesions, including capillary multiplication [27].

### 2.6. Statistical Analysis

Continuous variables are summarized as the median (interquartile range) and categorical variables as the number (percentage). Time to the first AE after diagnosis in BALF was obtained from the Kaplan–Meier survival curves in which the event of interest was AE instead of death. The clinical significance of each parameter as a predictor of AE-IIPs was determined using the univariate Cox proportional hazard analysis and multivariate Cox proportional hazard analysis with a stepwise method. The cutoff HS that predicted the AE-IIPs was determined by the analysis of receiver-operating characteristic curves for HS-positive cases. The values for the concordance statistics were compared using DeLong's method.

A *p* value of <0.05 was considered statistically significant. All statistical calculations were performed using SPSS, version 23 for Macintosh (IBM Corp., Armonk, NY, USA). DeLong's method was implemented using JMP v. 8.0 (SAS Institute Inc, Cary, NC, USA).

## 3. Results

### 3.1. Incidence of AE in IIPs

AE occurred in 45 cases (IPF, *n* = 28; non-IPF, *n* = 17) in 221 patients with IIPs (20.4%) during a median observation period of 1239 days. AE occurred in 14 (IPF, *n* = 13; nonspecific interstitial pneumonia (NSIP), *n* = 1) of the 53 cases diagnosed by SLB.

### 3.2. Distribution of Hemosiderin Score (HS)

The HS were positive in 24 (10.9%) of the 221 IIP cases. The median HS in these cases was 40.5 (interquartile range, 19.0‒64.5).

### 3.3. HS Cutoff Value That Predicted AE in HS-Positive Cases

The receiver-operating characteristic curve analysis identified the HS cutoff value that predicted AE to be 61.5. IIPs with an HS ≥ 61.5 were defined as the higher HS group (*n* = 7, 3.2%) and IIPs with an HS ≥ 0 and < 61.5 (*n* = 214, 96.8%) as a lower HS group.

### 3.4. Patient Demographics in the Lower and Higher HS Groups

The patient demographics are summarized in Table 1. BMI was significantly higher in the higher HS group than in the lower HS group. Other parameters at the time of diagnosis of IIPs and treatment for IIPs were similar between the two groups.

### 3.5. Relationship between HS and AEs in IIPs

**Figure 2:**
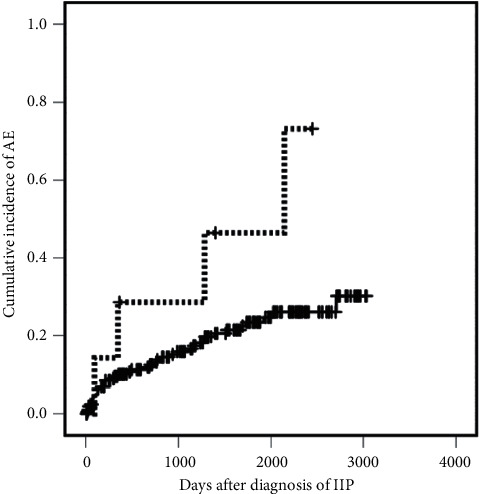
Kaplan–Maier curves showing the cumulative incidence of acute exacerbation (AE) in idiopathic interstitial pneumonias (IIPs). The median observation period from the date of bronchoalveolar lavage for the diagnosis of IIPs in total cases, IIPs with a lower hemosiderin score (HS), and the IIPs with a higher HS was 1239 days, 1214 days, and 1284 days, respectively. AE occurred significantly earlier in IIP cases with a higher HS (≥61.5; dotted line) than IIPs cases with a lower HS (<61.5; solid line, log-rank test, *p* = 0.026). The six-year occurrence rate of AE was 73.2% in IIPs with a higher HS and 26.1% in IIPs with a lower HS.

The incidence of AE was significantly higher in the higher HS group (4/7 cases, 57.1%) than in the lower HS group (41/214 cases, 19.2%) by Fisher's exact test (Table 1; *p* = 0.033). AEs also occurred significantly earlier in the higher HS group (*p* = 0.026, log-rank test; Figure 2). The severity of AE, indicated by the PaO_2_/FiO_2_ ratio and survival after AE, was similar between the higher and lower HS groups (Table 1; *p* = 1.000, Fisher's exact test and *p* = 0.121, log-rank test, respectively).

### 3.6. Predictors of AE in IIPs

#### 3.6.1. Cox Proportional Hazard Regression Analysis

The univariate analysis showed that a higher HS was a significant predictor of AE in addition to BMI, IPF, mMRC (≥2), %FVC, percent predicted diffusing capacity of carbon monoxide (%DLco), KL-6 level, and percentage of neutrophils in BALF (Table 2). The multivariate analysis of these parameters using the stepwise method revealed that a higher HS was a significant predictor of AE (Table 3).

#### 3.6.2. Predictive Models of Occurrence of AE-IIPs

The values for the C-statistics using IPF, mMRC, %FVC with (model 2) or without (model 1) the HS were 0.7932 (95% confidence interval [CI] 0.7170‒0.8351) and 0.7702 (95% CI 0.6877‒0.8361), respectively. There was no significant difference in the value of the C-statistics between the two models using DeLong's method (*p* = 0.1539; Table 4).

### 3.7. Histological Findings in SLB-Diagnosed Cases with a Higher HS

**Figure 3:**
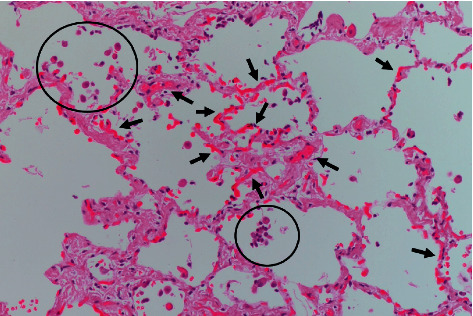
Histological findings of nonspecific interstitial pneumonia with a higher hemosiderin score. Aggregation of pigmented macrophages in alveolar spaces (circles) and capillary multiplication (arrow heads) was shown (Hematoxylin–Eosin staining).

Two of the SLB-diagnosed IIP cases (IPF, *n* = 1; NSIP, *n* = 1) had a higher HS (≥61.5; Table 1). Only the patient with NSIP experienced AE and died 9 days later. The histological findings for the two cases were re-evaluated. The aggregation of HLMs in the peripheral air spaces was observed in the patient with IPF; however, there was no marked capillary multiplication in this case. Both HLM aggregation and capillary multiplication were observed in the patient with NSIP but not always in close proximity (Figure 3). Stenosis of arterioles and venules was not observed in either case.

## 4. Discussion

This study is the first to investigate the ability of HLMs in BALF to predict AE-IIPs. In this study, multivariate Cox proportional hazard regression analysis identified a higher HLM level in BALF (HS ≥ 61.5) to be an independent predictor of AE-IIPs. However, the evaluation of the C-statistics showed that a higher HLM level in BALF could not improve the prediction of AE-IIPs and that its clinical impact was limited. There was no association between the severity of AE-IIPs and the HLM level in BALF or between the mortality and HLM level in BALF. The pathophysiology of HLMs and their relationship with AE-IIPs require further investigation.

Occult hemorrhage is an important endogenous cause of iron deposition in the lung and HLMs in the alveolar spaces and is associated with pulmonary hypertension [16, 17]. The Golde score was found to be significantly higher in patients with pulmonary veno-occlusive disease than in those with idiopathic pulmonary arterial hypertension [28]. Therefore, postcapillary vascular abnormalities are probably more important than arterial lesions in patients with occult alveolar hemorrhage.

The pathological examinations of vascular lesions in patients with an end-stage IPF who underwent lung transplantation found extensive vascular changes in the fibrotic areas in all cases but mainly in the muscular pulmonary arteries and arterioles [28]. Only mild changes were observed in architecturally preserved areas in these vessels. The main findings in these areas were those of the occlusion of pulmonary venules, which has been associated with alveolar capillary multiplication [27]. There have also been reports of similar changes in the capillaries, including increased microvessel density [29] or alveolar septal capillary density [16]. Iron deposition was found in interstitial and alveolar macrophages in the lung parenchyma of patients with the occlusion of venules and small pulmonary veins. Therefore, postcapillary vascular lesions may be associated with alveolar hemorrhage and iron deposition in patients with IPF. However, in a study about the time course of HLMs by Sherman et al., HLMs were rarely observed in the lung biopsy specimens obtained twelve days after acute hemorrhage [30]. Therefore, HLMs could have been cleared from the lung during this time. Alveolar hemorrhage associated with postcapillary vascular lesions in IPF possibly occurred continuously from the standpoint of transient HLM presence in the lung [30].

Kim et al. evaluated the density of alveolar septal capillaries in the nonfibrotic areas of SLB specimens. Univariate analysis revealed that the right ventricular systolic pressure measured by echocardiography was significantly associated with the alveolar septal capillary density and histologically scored iron deposition [16]. Iron deposition alone was a significant predictor of the right ventricular systolic pressure by multivariate analysis. Multivariate analysis in a study by Fukihara et al. also found that the ratio of HLMs to total macrophages, an indicator of iron deposition, was a significant predictor of pulmonary vascular resistance [17]. Therefore, HLMs reflect the microhemorrhage and are significantly correlated with the severity of pulmonary hypertension.

It has been hypothesized that the increased microvascular density or alveolar capillary multiplication in less fibrotic areas may represent a compensatory response to hypovascularity in the areas of dense fibrosis, i.e., honeycomb areas [29]. Ebina et al. suggested that alveolar-capillary multiplication in less fibrotic areas is caused by vascular endothelial growth factor (VEGF) produced by hyperplastic type II alveolar epithelial cells [31]. Le Cras et al. demonstrated in a transgenic mouse model that the overexpression of VEGF induced the hyperpermeability of the alveolar-capillary endothelium, which resulted in microhemorrhages and HLMs in the lung [32]. Therefore, VEGF-induced hyperpermeability of the alveolar-capillary endothelium may cause microhemorrhages in patients with IIP in the absence of pulmonary hypertension.

In this study, pulmonary hypertension was not evaluated by the right heart catheterization at the time of the diagnosis of IIP. Although a negative correlation between the right ventricular systolic pressure and % DLco has been reported [33], there was no significant deterioration of %DLco in our higher HS group at the time of BALF sampling, unlike in the lower HS group. Therefore, our patients with a higher HS did not necessarily have pulmonary hypertension, and occult hemorrhages may have occurred independently. In some patients with a higher HS, the hyperpermeability caused by intra-alveolar VEGF may have been associated with the occurrence of HLMs and occult hemorrhage.

The reason for the increased frequency of AE in our patients with a higher HS is unclear. The generation of iron-dependent reactive oxygen species (ROS) is a key feature in iron overload fibrosing diseases and the experimental models of pulmonary fibrosis [34]. It results in damage to the epithelial and endothelial cells [35, 36], which leads to chronic pulmonary fibrosis. Moreover, the HS in BALF from the patients with IPF reportedly correlates with iron deposition in the alveolar spaces and ROS levels [15]. Recently, Lee et al. reported that murine alveolar macrophages cultured with iron molecules were converted to HLMs and generated more ROS [37].

AE-IPF and ARDS share many clinical and pathological features [7, 38]. Hence, the uncontrolled inflammation, activation of coagulation pathways, and altered permeability of the endothelial and epithelial alveolar barriers observed in ARDS [39] are also pathophysiologically important in AE-IPF and AE-IIPs. Furthermore, ROS generated by inflammation and various stimuli are associated with increased permeability in ARDS [40]. It has been reported that the blood levels of free radicals in AE-IPF are higher than those in stable IPF [41]. Thus, the generation of ROS might be more strongly augmented by various stimuli in IPF with a higher HS. Therefore, both ROS-induced epithelial and endothelial cell damage and hyperpermeability may lead to AE in patients with IIP and a higher HS.

Alveolar-capillary multiplication and increased local VEGF levels may be associated with AE. The proliferating capillary cells can be injured more easily by ROS and other stimuli. This type of alveolar injury induces inflammation and hyperpermeability along with the increased production of VEGF in the alveolar epithelial cells, whereby new radiological shadows appear on nonhoneycomb areas in the lung with IPF.

In our study, BMI was significantly higher in the higher HS group. Higher BMI is a risk factor for AE-IPF [18], AE-IIP [9], and ARDS [42]. Furthermore, significantly elevated serum VEGF levels have been identified in patients with metabolic syndrome [43], suggesting that a systemic metabolic factor might induce VEGF, which is associated with a higher HS and increased likelihood of AE-IIPs.

This study had several limitations. Firstly, it had a retrospective, single-center design. Secondly, the group with a higher HS was too small for the use of C-statistics to confirm that the HS improved our ability to predict AE-IIPs. However, the multivariate Cox proportional hazard regression analysis showed that a higher HS was an independent predictor of AE in patients with IIP. Patients with IIP and a higher HS require careful monitoring for an early diagnosis of AE. Thirdly, although we hypothesized that capillary multiplication would be associated with the presence of HLMs, we could not confirm this association in two patients with a higher HS in whom the diagnosis of IIP was made by SLB. Further studies are needed to confirm this association. The fourth point is that IPF was diagnosed according to the 2011 guidelines [2]. We have already published the prognosis and occurrence of AE for 2005–2009 for our patients with IIP [9], most of whom were included in the present study. Therefore, we did not reevaluate the diagnosis of IPF in these patients according to the 2018 guidelines [44].

## 5. Conclusions

A higher HS is a significant predictor of AE-IIPs but does not improve the ability of other parameters to predict AE. Nevertheless, patients with IIP who have a higher HS in BALF required careful monitoring to avoid AE and to be able to treat AE as early as possible after its onset.

## Figures and Tables

**Table 1:** Patient demographics.

Parameters	Lower HS (*n* = 214)	Higher HS (*n* = 7)	*p* value
Sex, male/female	150/64	6/1	0.677
Age, yrs	69 (62–74)	62 (56–74)	0.185
BMI, kg/m^2^	24.0 (21.5–26.3)	28.0 (24.5–31.6)	0.024
Smoking, CS or ES/NS	159/55	7/0	0.197
Smoking CS/ES/NS	39/120/55	1/6/0	0.319
Iron dust exposure, yes/no	10/204	2/5	0.807
Autoantibody^*∗*^, yes/no	26/188	0/7	1.000
Diagnosis, IPF/non-IPF	84/130	3/4	1.000
HRCT, UIP/possible/inconsistent	56/96/62	3/1/3	0.291
SLB, yes/no	51/163	2/5	0.674
SLB-diagnosed cases			0.345
IPF	43	1	
NSIP	7	1	
LIP	1	0	
mMRC, < 2/≥ 2	137/77	6/1	0.426
%FVC, %	78.7 (64.6–93.9)	103.5 (70.5–115.2)	0.115
%DLco, %	52.3 (35.6–67.2)	50.9 (39.6–78.9)	0.766
KL-6, ×100 U/mL	8.95 (5.47–15.03)	7.90 (3.81–9.52)	0.271
SP-D, ×10 ng/mL	17.3 (9.8–26.6)	18.8 (14.6–24.9)	0.643
Neu in BAL, %	2.2 (0.8–7.2)	2.40 (0.5–4.6)	0.835
PT	1.01 (0.97–1.06)^†^	0.98 (0.95–1.84)^¶^	0.771
APTT	28.0 (25.6–30.4) ^‡^	27.8 (25.0–36.6)^¶^	0.478
Fibrinogen	308.3 (270.0–382.4) ^§^	286.0 (267.5–383.7)^¶^	0.845
Prednisolone use before AE, yes/no	55/159	0/7	0.197
Immunosuppressant before AE, yes/no	24/190	2/5	0.193
Observation period^#^, days	1214 (363-2028)	1284 (346-2144)	0.990
Occurrence of AE, yes/no	41/173	4/3	0.033
PaO_2_/FiO_2_ ratio at AE, ≤200/>200	25/16	2/2	1.000
AE-occurred cases/IIP diagnosis			
IPF	26/84	2/3	
NSIP	0/7	1/1	
LIP	0/1	0/0	
Non-IPF w.o. SLB	15/122	1/3	
Median survival days after AE^*∗∗*^	34	549	0.121

Abbreviations: AE, acute exacerbation; APTT, activated partial thromboplastin time; BAL, bronchoalveolar lavage; BMI, body mass index; CS, current smoker; DLco, diffusing capacity of carbon monoxide; ES, ex-smoker; FVC, forced vital capacity; HRCT, high-resolution computed tomography; HS, hemosiderin score; IIPs, idiopathic interstitial pneumonias; IPF, idiopathic pulmonary fibrosis; KL-6, Krebs von den Lungen-6; LIP, lymphocytic interstitial pneumonia; Neu, neutrophils; NS, nonsmoker; mMRC, modified Medical Research Council Score for shortness of breath; NSIP, nonspecific interstitial pneumonia; PT, prothrombin time; SLB, surgical lung biopsy; SP-D, surfactant protein-D; UIP, usual interstitial pneumonia. Data are presented as median (IQR) for continuous variables or as the number for categorical variables. Continuous variables were compared with Wilcoxon rank sum test and categorical variables were Fisher's exact test. ^*∗*^Antinuclear antibody (*n* = 5), rheumatoid factor (*n* = 5), anticyclic citrullinated peptide antibody (*n* = 4), myeloperoxidase-antineutrophil cytoplasmic antibody (*n* = 3), anticentromere antibody (*n* = 3), antiU1-ribonucleoprotein antibody (*n* = 3), antiRo/SSA antibody (*n* = 3), antiaminoacyl-tRNA synthetases antibody (*n* = 2), antidouble-strand deoxy nucleic acid antibody (*n* = 1), anti-La/SSB antibody (*n* = 1). Coagulation-related tests were performed: ^†^*n* = 220, ^‡^*n* = 219, ^§^*n* = 175, ^¶^*n* = 7. ^#^From diagnosis to onset of AE in AE-occurred cases or last follow-up in the other cases. ^*∗∗*^Survival of higher and lower HS groups were similar by log-rank test.

**Table 2:** Predictors of AE in IIPs (univariate Cox proportional hazard regression analysis).

Parameters	HR	95% CI	*p* value
Sex, Male vs. Female	1.571	0.776‒3.180	0.210
Age	1.028	0.992‒1.065	0.129
BMI	1.105	1.010‒1.210	0.030
Smoking, CS or ES vs. NS	1.100	0.556‒2.177	0.785
Iron dust exposure, yes vs. no	1.157	0.358‒3.737	0.807
Autoantibody, yes/no	0.267	0.065‒1.106	0.069
IPF vs. Non-IPF	2.745	1.499‒5.028	0.001
mMRC, ≥2 vs. <2	4.208	2.305‒7.682	< 0.001
%FVC	0.963	0.948‒0.978	< 0.001
%DLco	0.957	0.940‒0.975	< 0.001
KL-6, ×100 U/mL	1.031	1.004‒1.058	0.022
SP-D, ×10 ng/mL	1.005	0.998‒1.012	0.125
Neu in BAL, (%)	1.031	1.003‒1.059	0.027
HS, ≥61.5 vs. <61.5	3.026	1.082‒8.462	0.035

Abbreviations: AE, acute exacerbation; BAL, bronchoalveolar lavage; BMI, body mass index; CI, confidence interval; CS, current smoker; DLco, diffusing capacity of carbon monoxide; ES, ex-smoker; FVC, forced vital capacity; HR, hazard ratio; HS, hemosiderin score; IIPs, idiopathic interstitial pneumonias; IPF, idiopathic pulmonary fibrosis; KL-6, Krebs von den Lungen-6; mMRC, modified Medical Research Council Score for shortness of breath; Neu, neutrophils; NS, nonsmoker; SP-D, surfactant protein-D.

**Table 3:** Predictive factors of AE in IIPs (multivariate Cox proportional hazard regression analysis).

Parameters	HR	95% CI	*p* value
mMRC, ≥2 vs. <2	3.345	1.690‒6.623	0.001
HS, ≥61.5 vs. <61.5	5.649	1.919‒16.624	0.002
IPF vs. Non-IPF	3.268	1.754‒6.098	< 0.001
%FVC	0.964	0.945‒0.984	0.001

Abbreviations: AE, acute exacerbation; CI, confidence interval; FVC, forced vital capacity; HR, hazard ratio; HS, hemosiderin score; IIPs, idiopathic interstitial pneumonias; IPF, idiopathic pulmonary fibrosis; mMRC, modified Medical Research Council Score for shortness of breath. Multivariate Cox proportional hazard regression analysis with a stepwise selection method using significant parameters shown in Table 2 was performed.

**Table 4:** Predictive models of occurrence of AE-IIPs with/without hemosiderin score.

	Parameters	C-statistics	95% CI
Model 1:	IPF vs. Non-IPF mMRC, ≥2 vs. <2 %FVC	0.7702	0.6877‒0.8361
Model 2:	IPF vs. Non-IPF mMRC, ≥2 vs. <2 %FVC HS, ≥61.5 vs. <61.5	0.7932	0.7170‒0.8531

Abbreviations: CI, confidence interval; FVC, forced vital capacity; HS, hemosiderin score; IPF, idiopathic pulmonary fibrosis; mMRC, modified Medical Research Council Score for shortness of breath. Difference in the C-statistics of the two models was not significant using DeLong's method (*p* = 0.1539).

## Data Availability

The datasets used and/or analyzed in this study are available from the corresponding author upon reasonable request.
